# Study of the Influence of Thermomechanical Treatment on the Structure and Properties of Zircalloy-4 Alloy

**DOI:** 10.3390/ma19091711

**Published:** 2026-04-23

**Authors:** Fedor Popov, Anna Kawalek, Kirill Ozhmegov, Nikita Lutchenko, Evgeniy Panin, Sergey Lezhnev, Alexandr Arbuz

**Affiliations:** 1 Core Facilities and HPC, AEO Nazarbayev University, 53 Kabanbay Batyr Ave., Astana 010000, Kazakhstan; 2 Metal Forming Department, Czestochowa University of Technology, ul. J.H. Dabrowskiego 69, 42-201 Czestochowa, Poland; anna.kawalek@pcz.pl (A.K.);; 3 Metal Forming Department, Karaganda Industrial University, 30 Republic Ave., Temirtau 101400, Kazakhstan; 4 Metallurgy and Mining Department, Rudny Industrial University, 50 let Oktyabrya Str. 38, Rudny 111500, Kazakhstan

**Keywords:** zirconium-based alloy, rheological properties, dilatometry, electron backscatter diffraction

## Abstract

The Zircaloy-4 alloy is a key structural material for nuclear reactor cores. However, its behavior under warm deformation conditions and during phase transformations requires in-depth investigation to improve technologies for producing ultrafine-grained (UFG) structures using severe plastic deformation methods. This work presents a comprehensive study of the rheological properties, phase stability, and microstructural evolution of the alloy in the temperature range from 20 to 950 °C at strain rates of 0.5 and 15 s^−1^. The experimental part included plastometric testing, dilatometric analysis, and microstructural characterization. It was established that the optimal window for plastic deformation corresponds to warm deformation at 650 °C. Dilatometric analysis confirmed that heating to 650 °C ensures the preservation of a stable initial α-phase structure, since the formation of secondary phases and the α→β transformation are initiated at higher temperatures, namely 694 °C (onset) and 847 °C (completion). At 650 °C, the deformation resistance decreases by approximately 70% compared to cold processing, while the strain-rate sensitivity of the flow stress is minimized. EBSD analysis showed that deformation under these conditions leads to intensive grain fragmentation via mechanisms of dynamic recovery and the initial stages of continuous dynamic recrystallization. The decisive role of the kinetic factor was demonstrated: reducing the strain rate to 0.5 s^−1^ promotes the formation of a finer and more homogeneous grain structure. In contrast, high strain-rate deformation (15 s^−1^) results in coarser grains and increased non-relaxed intragranular residual stresses. The obtained results provide a physical basis for optimizing thermomechanical processing regimes and can be used to produce UFG structures in zirconium alloys without the risk of phase degradation.

## 1. Introduction

The Zircaloy-4 zirconium alloy, with a chemical composition of Zr–1.5Sn–0.2Fe–0.1Cr, belongs to the group of structural materials used in nuclear power engineering. Compared to the Zircaloy-2 alloy, it does not contain nickel, which reduces hydrogen absorption under reactor operating conditions and limits the risk of hydrogen embrittlement. Due to its favorable performance properties, such as corrosion resistance in water and steam environments, structural stability at high temperatures, and resistance to intense neutron radiation, this alloy is used to manufacture fuel cladding and fuel assembly (FA) components in PWR (Pressurized Water Reactor) and VVER (Water-Water Energetic Reactor) reactors [1]. This alloy is characterized by a low neutron absorption cross-section, which is crucial in power reactors as it minimizes the loss of neutrons necessary to sustain the chain reaction. Additionally, it forms a compact ZrO_2_ oxide layer on its surface, which improves corrosion resistance and limits hydrogen diffusion into the material [2,3,4].

These properties were sufficient for the use of Zircaloy-4 as the primary structural material in PWR and VVER reactor cores. However, modern reactors operate under more demanding conditions, characterized by high temperatures, great pressures typically 360 °C and 15.5 MPa [5], and a more intense neutron flux causing increased damage (6.2–6.5 dpa at 10^24^/m^2^ fluence, E > 1 MeV) [6]. Such conditions accelerate corrosion processes, material oxidation, and radiation-induced creep and growth. As a result, the requirements for structural materials have increased; they must demonstrate enhanced corrosion resistance [4], high-temperature strength, and stability under intense radiation conditions. Furthermore, growing demands regarding fuel efficiency and emergency safety are exceeding the capability limits of traditional Zircaloy-4 [7,8].

New materials capable of meeting the requirements of modern nuclear reactors can be obtained, for example, by modifying the chemical composition of the alloy. However, changing the chemical composition is very difficult because each component and its percentage content in the alloy have a direct impact on the dynamics of fission processes—even minor changes can alter the reaction kinetics, phase stability, or mechanical properties of the material, and the introduction of new elements may result in unpredictable side effects, such as structural defects, decreased corrosion resistance, or a change in the melting point. Therefore, it is important to look for other ways to improve the material’s properties without modifying its chemical composition. One such method is grain refinement to obtain ultrafine-grained (UFG) structures. UFG structures with grain sizes less than 1000 nm are characterized by increased strength by a factor of 1.5–2 while maintaining ductility, improved creep resistance, and lower susceptibility to defects caused by neutron radiation, making them particularly attractive for structural materials used in nuclear reactors [9,10,11]. These properties are explained by the fact that, as the grain size decreases, the total length of grain boundaries increases dramatically, and they begin to make a significant contribution to the overall properties of the material [12].

One of the effective methods of grain boundary engineering is the formation of UFG structures by Severe Plastic Deformation (SPD) [13]. The formation of such a structure requires not only a high level of total strain (6–8 mm/mm) under conditions of non-monotonic metal flow, but also the provision of a reduced deformation temperature. It is recommended to use temperatures below 0.4 of the melting temperature (T_m_) in order to suppress recrystallization. At the same time, deformation in the cold state carries a high risk of material failure due to a dramatic decrease in ductility. Therefore, for successful cold and cryogenic deformation without material fracture, a condition of all-around hydrostatic compression with a pressure of about 1 GPa or higher is required [14].

Such conditions can only be achieved using specialized laboratory deformation techniques, such as High-Pressure Torsion (HPT) [15,16]. Therefore, for more practical scenarios involving large plastic deformation—such as radial-shear rolling [17]—it is necessary to find a thermal compromise between minimizing recrystallization-driven grain growth and maintaining sufficient ductility for material flow. This approach has yielded promising results in the Zr–1%Nb alloy [18].

Despite the widespread use of the Zircaloy-4 alloy, its behavior under hot plastic deformation conditions during complex thermomechanical cycles has still not been fully investigated. A particularly important issue remains the characterization of the α–β phase transition, the course of which in this material is of key importance for shaping the microstructure and mechanical properties of the final products. The boundaries of the α–β phase transition in Zircaloy-4 are not constant and show a clear dependence on heating parameters, such as the heating rate, soaking time, and the history of prior plastic deformation. This variability directly affects the kinetics of phase transformations, the fraction and morphology of the β phase, and consequently the stability and homogeneity of the microstructure. Under rapid heating conditions, the transformation start and finish temperatures may shift towards higher values, which is related to diffusion limitations and the non-equilibrium nature of the process. The data available in the literature regarding the effect of high heating rates on the position of the α–β transformation temperatures are limited in scope and do not provide unequivocal conclusions [19,20]. Therefore, it is purposeful to conduct precise dilatometric studies that allow for the unambiguous determination of the start and finish temperatures of the phase transformation, as well as the quantitative assessment of their changes depending on the heating conditions.

Differences in the material’s behavior at various strain rates are of crucial importance for describing its rheological behavior under warm and hot deformation conditions. It has been shown in the literature that the course of plastic deformation of zirconium alloys is strictly dependent on temperature and strain rate, which is directly reflected in an increase or decrease in the flow stress values [21,22]. At lower temperatures and higher strain rates, an increase in flow stress is observed, which is associated with the accumulation of crystal lattice defects, while an increase in temperature promotes its reduction due to the intensification of dynamic recovery and recrystallization processes [23,24]. Moreover, the microstructure and crystallographic texture significantly affect the alloy’s resistance to creep and radiation degradation, which is of key importance for materials intended for nuclear applications [25,26,27]. Consequently, it is necessary to conduct rheological studies enabling the determination of the dependence of flow stress on temperature and strain rate. The analysis of flow curves will allow for determining the range of deformation parameters for the Zircaloy-4 alloy in which the material maintains microstructural stability without excessive grain growth.

The aim of this study was a comprehensive characterization of the thermomechanical behavior of the Zircaloy-4 alloy under Severe Plastic Deformation (SPD) conditions.

The scope of the research included determining the temperature ranges of the α–β phase transition, taking into account heating and cooling kinetics, and determining the dependence of flow stress on temperature and strain rate over a wide range of temperatures (20–950 °C) and strain rates of 0.5 s^−1^ and 15 s^−1^. The conducted analysis of microstructural changes will make it possible to identify the optimal range of plastic deformation conditions ensuring the achievement of a stable ultrafine-grained (UFG) structure while maintaining the required performance properties. The deformation parameters were refined within the most favorable temperature range of warm deformation (550–650 °C). Detailed EBSD maps were obtained for all cases.

## 2. Materials and Methods

One of the most common zirconium alloys, Zircaloy-4, was selected as the material for the research. Its chemical composition consists of a zirconium base with alloying additions shown in Table 1. This alloy is widely used in the manufacture of nuclear fuel cladding tubes and end plugs for fuel elements in thermal neutron reactors (specifically, PWR and CANDU types). For this study, the material was purchased in the form of a 12 mm diameter rolled round bar.

To study the patterns of metal flow in relation to the temperature and strain rate conditions of radial-shear rolling, plastometric tests were conducted. The research was carried out using the uniaxial compression method on cylindrical specimens with a working section diameter of 10 mm. The experiments covered a temperature range from 20 to 950 °C and strain rates from 0.5 to 15 s^−1^.

**Figure 1:**
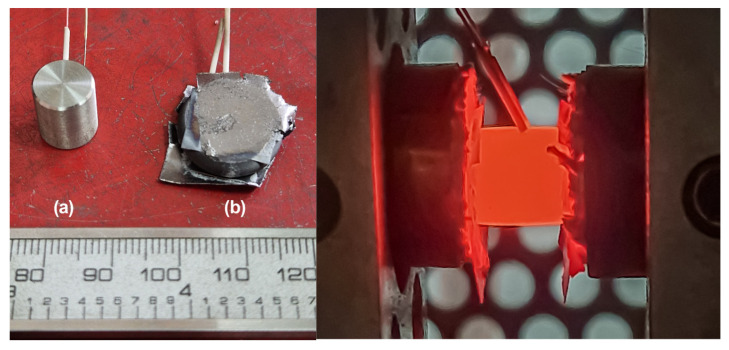
Zircaloy-4 alloy specimens before (**a**) and after (**b**) plastometric testing (**left**) and the high-temperature testing process (**right**).

The tests were performed under continuous loading conditions on a specialized Gleeble 3800 plastometric testing system (Gleeble Systems, Poestenkill, NY, USA) using the Pocket Jaw module (Figure 1). This equipment allows for the physical simulation of conditions for various metal forming processes and enables experiments with high precision in data recording. During deformation, temperature fluctuations did not exceed ±1.0 °C, the load measurement error was ±1.0 kg per 1 ton, and the displacement accuracy of the working crosshead was within ±0.01 mm. The remaining parameters were calculated as derivatives of these base quantities.

Temperature control directly within the testing zone was achieved using a chromel-copel thermocouple. It was welded to the central section of the specimen utilizing the specialized Thermocouple Welder unit supplied with the Gleeble 3800 system. To minimize contact friction between the specimen and the tooling, thin graphite foils were applied. Furthermore, after each testing cycle, the working anvils (ISO-T model) were additionally coated with OKS255 graphite lubricant. The dimensions of the deformed samples after plastometry are 16–20 mm in diameter with a thickness of 6–4 mm, respectively (Figure 1 left (b)).

Dilatometric studies make it possible to obtain data on the dynamics of structural and phase transformations occurring during heating. Based on these data, taking into account the thermal stability of the phase composition, the optimal heating temperature of the workpieces before intensive plastic deformation will be determined.

**Figure 2:**
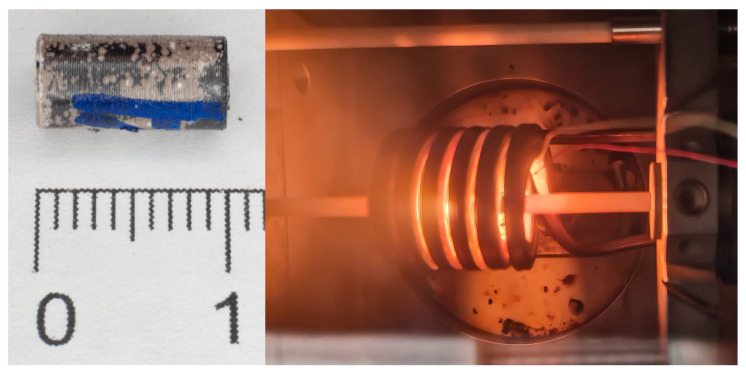
Zircaloy-4 alloy specimen after testing (**left**) and the testing process in the DIL 805A/D quenching and deformation dilatometer (**right**).

The experiments were performed using a high-precision DIL 805A/D quenching and deformation dilatometer (TA Instruments, New Castle, DE, USA) (Figure 2). Cylindrical specimens with a working section diameter of 5 mm were used as the objects of study.

The tests were conducted according to a strictly defined thermokinetic cycle consisting of three consecutive stages. In the first stage, controlled heating of the specimens was carried out at a rate of 10 to 20 °C per minute until the target test temperature of 1100 °C was reached. This was followed by a brief isothermal hold at this temperature for 30 s, which was necessary to ensure uniform heating of the material throughout the entire volume of the specimen. In the third stage of the cycle, accelerated cooling was performed at a rate of 30 °C per second in order to retain the high-temperature structural state of the alloy.

Microstructural analysis of the deformed specimens was performed by electron backscatter diffraction (EBSD) using a Helios-5CX dual-beam scanning electron microscope (FIB/SEM) (Thermo Fisher, Bleiswijk, Netherlands) equipped with an EDAX Velocity Ultra EBSD system (EDAX, Mahwah, NJ, USA). Sample preparation for the examination was carried out on a Brilliant-220 (QATM, Mammelzen, Germany) precision cutting machine. Plates 1.5 mm thick were cut from the cross section of the deformed sample. To minimize thermomechanical damage to the microstructure, the cutting was performed under intensive water cooling using coarse-grained abrasive discs at a feed rate of 5 µm/s and a rotation speed of 700 rpm. The final surface preparation of the cross-sections (removal of the deformed surface layer) was conducted by electrolytic polishing on LectroPol-5 system (Struers, Ballerup, Denmark) with the A3 electrolyte (600 mL Methanol, 360 mL Butylcellosolve, 60 mL Perchloric Acid). The prepared samples were scanned with a step size of 0.1 microns at a magnification of 1500×, 3000× and 8000×. The acquired crystallographic data arrays were used to construct inverse pole figure (IPF) maps, kernel average misorientation (KAM) maps, and direct pole figures. Specifically, the EBSD analysis was conducted on samples in their initial state (20 °C), as well as on specimens deformed at strain rates of 0.5 s^−1^ and 15 s^−1^ at temperatures of 200, 550, 580, 650, 750, and 950 °C.

## 3. Results

### 3.1. Plastometry

A comprehensive evaluation of the thermomechanical behavior of the Zircaloy-4 alloy primarily requires an analysis of its rheological characteristics. The material’s response to changes in temperature and strain rate parameters directly determines the stability of plastic flow, the nature of microstructural evolution, and, consequently, the level of the final physical and mechanical properties of the manufactured products. In this regard, the initial stage of this research focused on studying the patterns of strain hardening and dynamic softening of the alloy under various thermomechanical loading conditions.

**Figure 3:**
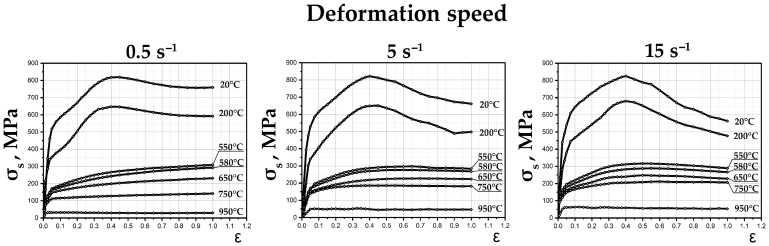
Strain hardening curves of the Zircaloy-4 alloy during compression tests on the Gleeble 3800 plastometer.

Figure 3 presents the true flow stress curves (strain hardening curves) for the Zircaloy-4 alloy, obtained during continuous uniaxial compression of the specimens on the Gleeble 3800 plastometer over a temperature range of 20 to 950 °C and at strain rates from 0.1 to 15 s^−1^. Graphs in their original format can be found in Supplementary Materials as Figure S1.

An analysis of the curves shows that at a test temperature of 20 °C, the strain hardening coefficient of the alloy decreases noticeably as the strain increases, since the multiple slip stage transitions into the parabolic section of the curve. In the region of high strains (ε = 0.3–0.4), the influence of the strain rate becomes marginal. This is attributed to the thermal effect of plastic deformation (adiabatic heating), which at a rate of 15 s^−1^ can reach a value of ΔT ≈ 150 °C. Upon reaching a true strain of ε > 0.4, a decrease in flow stress is observed for all strain rates. This effect is explained by a geometric factor—the macrolocalization of plastic deformation (intensive barreling on the lateral surface of the specimen). Concurrently, increasing the strain rate to 15 s^−1^ triggers an earlier onset of localization. Thus, conducting deformation at high rates at room temperature is undesirable due to a significant uncontrolled thermal effect and the early loss of metal flow stability.

Increasing the deformation temperature to 200 °C leads to an expected decrease in deformation resistance (σ_s_) by approximately 18%. At the same time, no significant change in the overall nature of the flow curves is observed. It is only worth noting a slight increase in the strain rate sensitivity of the flow stress at 15 s^−1^. Decreasing the strain rate to 0.5 s^−1^ delays the onset of deformation localization (by a value of 0.05) compared to tests at room temperature. The use of this temperature regime is of practical interest from the perspective of reducing the power and force parameters of the process, with a complete absence of risks associated with active gas saturation of the alloy.

Special attention in this study was given to the temperature range of 550–650 °C (≈0.3–0.35 T_m_), where investigations were conducted with a reduced temperature step. This range is considered the most promising for implementing warm severe plastic deformation (SPD) regimes for several reasons:

- Firstly, it is below the temperature of the α→β phase transition, which allows deformation to be applied at the final stages of the technological cycle without the risk of degrading the formed microstructure or deteriorating the operational properties of the finished product;
- Secondly, this is a temperature range of active development of dynamic recovery processes and the onset of partial/complete recrystallization, which significantly reduces the metal’s resistance to deformation and increases its ductility;
- Thirdly, processing within this range minimizes the intensity of surface gas saturation, which simplifies or eliminates the need for complex protective coatings.

Within the specified temperature range, the general family of flow curves maintains a similar character. The flow stress (σ_s_) consistently decreases: during the transition from 550 to 580 °C, the drop is about 12%, and from 550 to 650 °C, it is approximately 25%. Compared to cold deformation (20 °C), the flow resistance drops significantly—by roughly 70%. The σ_s_–ε curves obtained at rates of 5 and 15 s^−1^ exhibit a pronounced dome-shaped character: the stress reaches a peak value, followed by a softening stage, indicating that dynamic softening processes begin to prevail over strain hardening processes.

At a temperature of 650 °C and a strain rate of 5 s^−1^, an extended region of steady-state flow is recorded (where the curve reaches a plateau), which is a clear sign of the intensive occurrence of dynamic recovery processes. It is known that in the stages preceding steady-state flow, a cellular dislocation structure forms in the metal. The critical strain required for its formation is lower at higher temperatures and lower strain rates. During the steady-state flow stage (in the region of high strains), the structure acquires a more equilibrium character with a comparatively low scalar dislocation density.

It is interesting to note that the flow curves obtained at a rate of 0.5 s^−1^ intersect the curves corresponding to higher strain rates at certain stages, and at 650 °C, the difference between them becomes minimal. Considering that severe plastic deformation processes of cylindrical billets are often characterized by a significant strain rate gradient across the cross-section (from the center to the periphery), minimizing the strain rate sensitivity of the flow stress at 650 °C contributes to reducing the level of residual internal stresses. Consequently, this temperature is thermomechanically optimal for heating the alloy prior to severe deformation.

In the hot deformation temperature range (650–950 °C) and the strain rate range of 0.5–15 s^−1^, the stress peak shifts to the region of low strains and becomes less pronounced. A steady-state flow stage is clearly recorded on the curves, the extent of which consistently increases with rising temperature. At the maximum study temperature (950 °C) on the curves obtained at rates of 5 and 15 s^−1^, the nature of the stress changes unequivocally indicates the active development of dynamic recrystallization processes within the material.

### 3.2. Dilatometry

To better understand the thermomechanical behavior of the Zircaloy-4 alloy and to provide a physical rationale for the selected deformation temperature regimes, the analysis of its rheological properties must be considered in close conjunction with the structural and phase stability of the material. It is known that the technological plasticity of zirconium alloys and the evolution of their microstructure critically depend on the occurrence of phase transformations—primarily, the polymorphic α→β transition and the dissolution processes of secondary phases. In this regard, a dilatometric analysis of the alloy was conducted at specified heating and cooling rates.

According to the testing program, the studies were performed on specimens in an initial, fully recrystallized state with a grain size corresponding to 9–10 on the standard metallographic scale.

**Figure 4:**
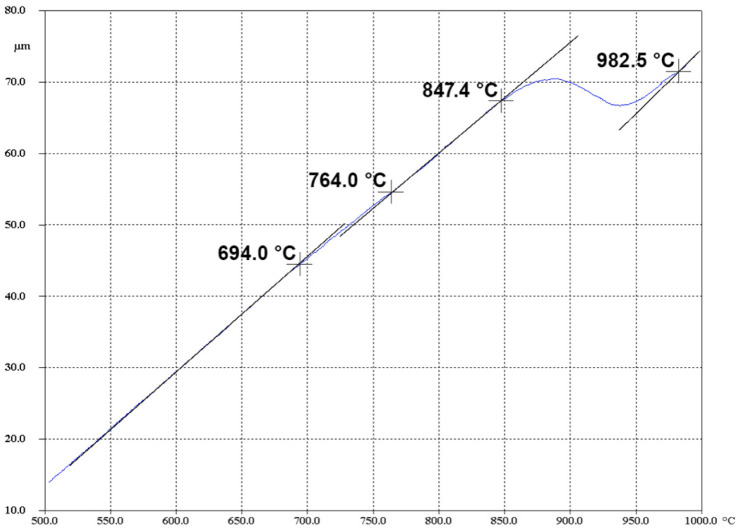
Dilatometric curve of the Zircaloy-4 alloy during specimen heating on the DIL 805A/D at a rate of 20 °C/s up to T = 1100 °C.

An analysis of the dilatometric heating curve (Figure 4) shows that in the temperature range from room temperature to 694 °C, monotonic thermal expansion of the specimen occurs. Upon reaching temperatures of 694 °C and 764 °C, a change in the slope angle is observed on the curve, which indicates the onset and completion of the dissolution of secondary β-phases. Further heating in the temperature range of 847–982.5 °C is accompanied by the polymorphic α→β transformation (the transition from a hexagonal close-packed to a body-centered cubic crystal lattice). The obtained temperature ranges for the phase transitions are in good agreement with the literature data [29].

**Figure 5:**
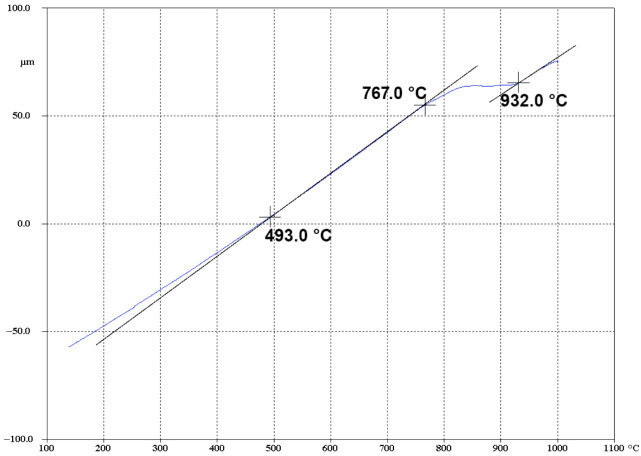
Dilatometric curve of the Zircaloy-4 alloy during specimen heating on the DIL 805A/D at a rate of 30 °C/s up to T = 1100 °C.

During the subsequent cooling of the metal at a specified rate (Figure 5), critical points are also clearly recorded on the dilatometric curve. The completion of the reverse polymorphic β→α transformation is registered at a temperature of 767 °C. Upon further cooling to 493 °C, the phase transition associated with the precipitation of dispersed secondary phase particles is completed in the Zircaloy-4 alloy.

Thus, the comprehensive analysis of the plastometric and dilatometric research results confirms that 650 °C is the optimal heating temperature for the billets prior to severe plastic deformation. This temperature regime not only ensures the high technological plasticity of the material and promotes a uniform stress distribution across the cross-section of the deformed billet, but it also securely remains below the temperature threshold for the onset of phase transformations. This makes it possible to preserve the stability of the initial α-phase microstructure immediately prior to the start of deformation.

### 3.3. EBSD

**Figure 6:**
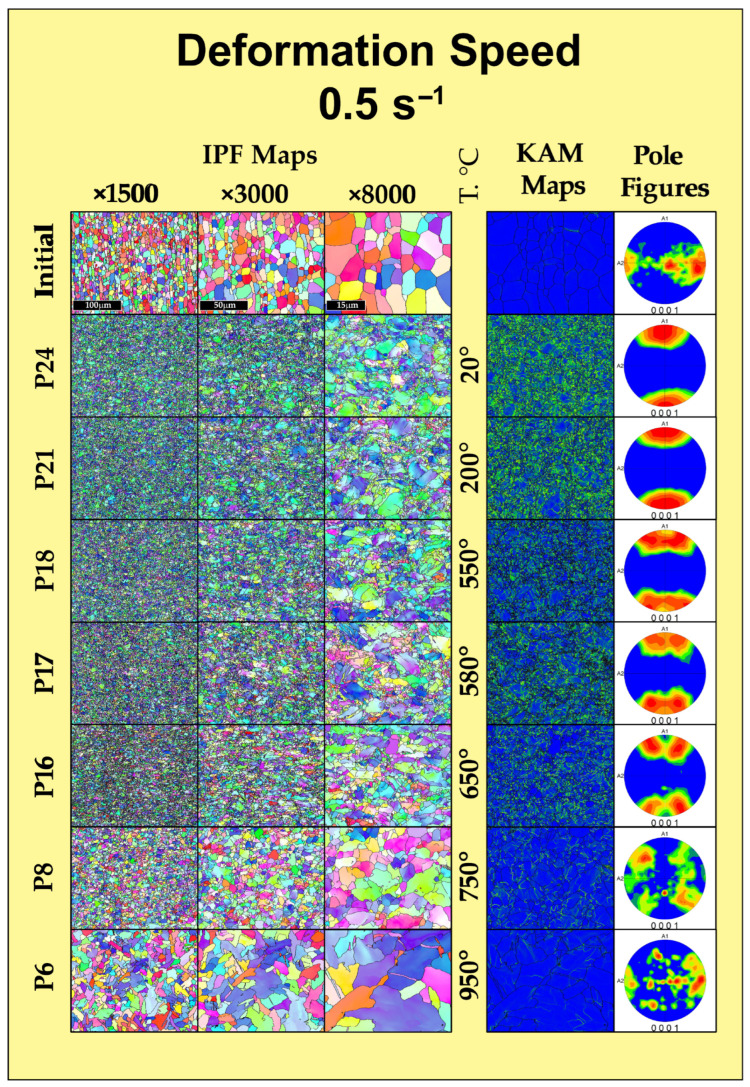
Inverse pole figure (IPF) orientation maps, kernel average misorientation (KAM) maps, and direct pole figures of the Zircaloy-4 alloy in the initial state and after deformation at various temperatures at a strain rate of 0.5 s^−1^.

**Figure 7:**
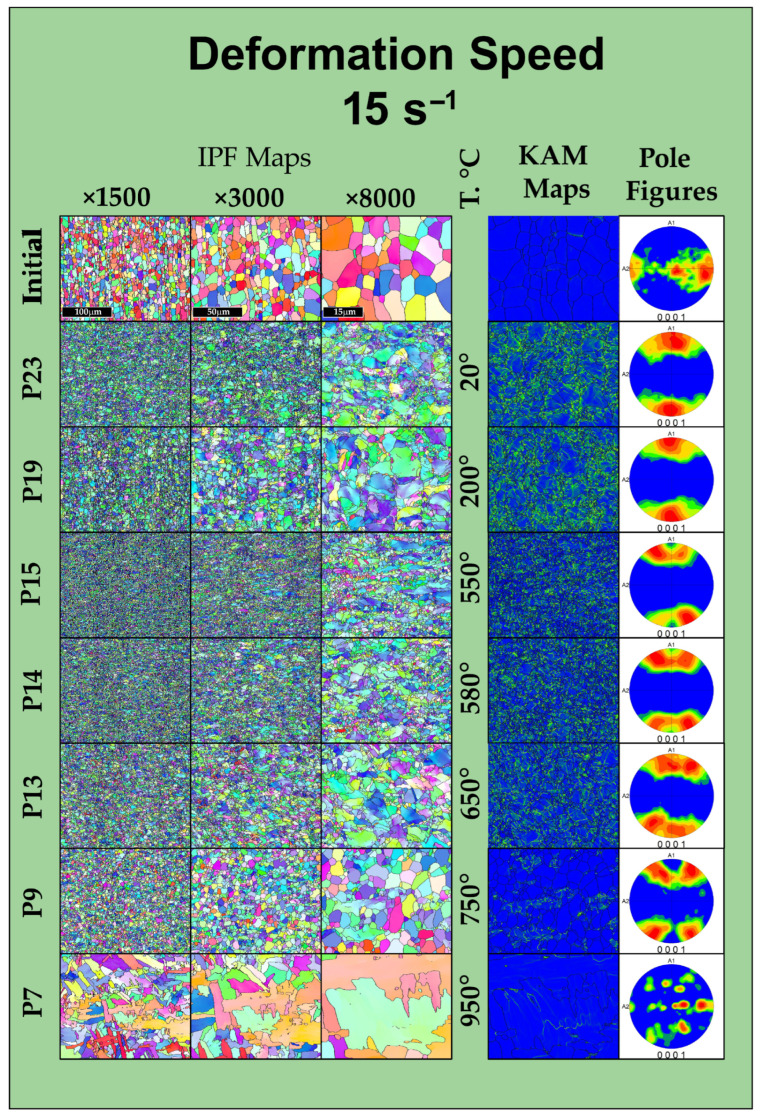
Inverse pole figure (IPF) orientation maps, kernel average misorientation (KAM) maps, and direct pole figures of the Zircaloy-4 alloy in the initial state and after deformation at various temperatures at a strain rate of 15 s^−1^.

To establish the relationship between the rheological behavior of the Zircaloy-4 alloy and its microstructural evolution, the deformed specimens were analyzed using electron backscatter diffraction (EBSD). The corresponding EBSD maps of the deformed specimens are presented in Figure 6 for a strain rate of 0.5 s^−1^ and in Figure 7 for a strain rate of 15 s^−1^. Figures in their original format can be found in Supplementary Materials as Figures S2 and S3.

Analysis of the initial state of the alloy prior to thermomechanical processing shows that the material is in a recrystallized state following preliminary deformation. The IPF maps demonstrate a homogeneous structure consisting predominantly of equiaxed α-phase grains with well-defined high-angle boundaries and sizes in the range of 8–10 μm. As seen on the pole figure, the crystallographic texture along the specimen axis has an appearance characteristic of the extrusion or rolling of a round billet. At the same time, the Kernel Average Misorientation (KAM) maps show the absence of residual stresses and a low dislocation density within the grain volume.

After deformation at low temperatures (20–200 °C), a significant change in the grain structure compared to the initial state is already observed. During plastic flow, the fragmentation of primary grains and the formation of a substructure with small grain sizes occur. Significant degrees of local misorientation (KAM) appear near the boundaries of the primary grains, associated with accumulated strain and stress. Regions with elevated local misorientation angles emerge on the KAM maps. This indicates a high density of dislocation pile-ups and the accumulation of deformation energy. According to the pole figures, the crystallographic texture changes in accordance with the uniaxial deformation experienced by the specimen. An increase in the fraction of low-angle boundaries while maintaining the elongated shape of the initial grains indicates the development of continuous dynamic recrystallization processes, which do not reach completion due to the relatively low temperatures.

In the warm deformation temperature range of 550–650 °C (specimens P18, P17, P16 for a rate of 0.5 s^−1^ and their corresponding specimens at 15 s^−1^), a more intense change in both the structure and grain size occurs, while the texture is preserved. After deformation, the structure acquires a heterogeneous appearance with a wider grain size distribution range (from 0.3 to 10 μm). The structure contains both coarse grains (comparable to the initial structure) and clusters of fine grains (0.3–2 μm). The preservation of a high defect density, according to the KAM maps, confirms the deformation potential in this range (specifically at 650 °C) for the formation of ultrafine-grained structures.

With an increase in the deformation temperature, the structure formation changes. Specimen P8, deformed at 750 °C, demonstrates a transitional state: due to the increased atomic mobility, dislocation migration processes are activated, leading to the development of recrystallization. This is evident from both the increase in grain size and the decrease in local misorientations on the KAM maps, alongside the coarsening of subgrains. A weaker texture can also be observed at this temperature. It should be noted that there is some difference in recrystallization for different strain rates. Specifically, at a rate of 0.5 s^−1^, a slightly larger increase in grain size and texture weakening are observed, whereas at 15 s^−1^, the grains remain more equiaxed while maintaining a high level of texture.

In the region of high-temperature deformation at 950 °C (specimen P6) and 1050 °C (specimen P4), the EBSD patterns change significantly. Intensive grain growth and the absence of texture are observed. A lamellar morphology is formed, which is a characteristic feature of the polymorphic α→β→α transformation. Deformation occurs in the β-region, after which the α-phase forms upon cooling. The KAM maps for these specimens demonstrate a complete relaxation of deformation stresses, which is typical for structures formed during cooling.

**Figure 8:**
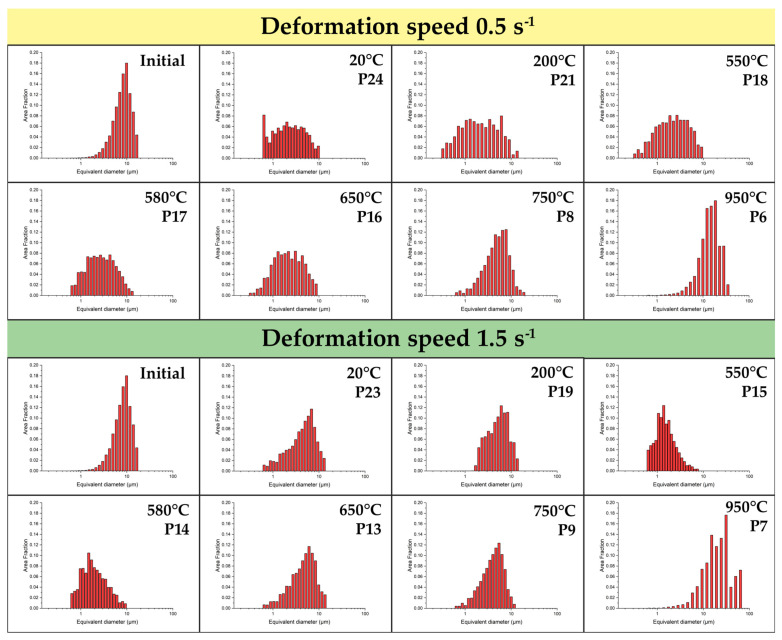
Grain size distribution histograms at a temperature of 650 °C and different strain rates: (**upper**)—0.5 s^−1^, (**lower**)—15 s^−1^.

A comparative analysis of the structures formed at rates of 0.5 s^−1^ and 15 s^−1^ at 650 °C allows evaluating the influence of the kinetic factor. An analysis of the grain size distribution histograms (Figure 8) confirms the influence of the kinetic factor on microstructural evolution: despite the same minimum recorded grain size (about 0.32 μm), high-rate deformation (15 s^−1^) leads to the formation of a coarser structure with a maximum grain size of up to 10.39 μm and a predominant grain area fraction at 5.0 μm. Decreasing the rate to 0.5 s^−1^ ensures the formation of a finer microstructure with a maximum size of 8.6 μm, a shift in the predominant area fraction to 3.1 μm, and a uniform grain distribution in the range of 1–6 μm. Specifically, at 650 °C, the average grain size is 5.28 ± 2.96 μm for the strain rate of 15 s^−1^, compared to a finer average size of 2.713 ± 1.928 μm at 0.5 s^−1^. The obtained statistical data prove that increasing the duration of thermomechanical exposure at 0.5 s^−1^ creates the necessary conditions for a more complete occurrence of continuous dynamic recrystallization processes, whereas at 15 s^−1^, this effect is reduced.

## 4. Discussion

A comprehensive analysis of rheology, phase stability, and microstructural evolution of the Zircaloy-4 alloy allows for the justification of the optimal temperature and strain rate parameters for its plastic deformation. The main technological objective when processing such alloys is to strike a balance: it is necessary to maximally reduce the material’s resistance to deformation in order to decrease the power and force parameters of the equipment, while simultaneously maintaining phase stability and ensuring the formation of an UFG structure.

The results of the plastometric tests demonstrate that conducting deformation at room temperature is technologically impractical. A high level of strain hardening and an intense thermal effect (adiabatic heating up to 150 °C at a rate of 15 s^−1^) provoke the early macrolocalization of plastic flow. In turn, transitioning to the high-temperature region (above 750 °C) resolves the problem of deformation resistance but leads to significant microstructural degradation. EBSD analysis data confirm that at 750 °C, collective recrystallization processes are activated, leading to grain coarsening. Upon reaching temperatures of 950–1050 °C, a complete transformation of the structure occurs: according to dilatometric data, the material transitions into the β-phase region, and during subsequent cooling, a coarse, enlarged structure is formed. Such a morphology, despite the complete relaxation of internal stresses, contradicts the objective of achieving a UFG state.

Against the backdrop of these limitations, the temperature range of 550–650 °C (warm deformation regime) emerges as the most promising technological window. Dilatometric studies confirm that up to 694 °C, the material maintains a stable α-phase structure, avoiding the dissolution of secondary phases and polymorphic transformations. This guarantees the predictability of structural changes during the deformation process.

The mechanical behavior of the alloy in this range is also optimal. At 650 °C, the deformation resistance decreases by approximately 70% compared to cold deformation. An important factor is the convergence of the flow curves obtained at different strain rates (0.5 and 15 s^−1^) specifically at the temperature of 650 °C. In actual processes of severe plastic deformation of cylindrical billets, a strain rate gradient across the cross-section is often present. Minimizing the strain rate sensitivity of the flow stress at 650 °C helps ensure the homogeneity of the stress–strain state and reduces the level of residual stress.

The microstructural mechanisms occurring at 650 °C fulfill the objective of structure refinement. EBSD maps record the intensive fragmentation of primary α-grains and the formation of a developed substructure. Unlike high-temperature deformation, high dislocation density is preserved here. This indicates that the primary mechanism of structural evolution is dynamic recovery combined with continuous dynamic recrystallization. The formed substructure, with its high accumulated deformation energy, is essential for the formation of a homogeneous ultrafine-grained state in subsequent processing stages.

The influence of the kinetic factor (strain rate) in this temperature window has a dual nature. On the one hand, increasing the rate to 15 s^−1^ limits the migration mobility of boundaries and allows for the achievement of a more dispersed structure. On the other hand, a sharp reduction in deformation time restricts the completeness of dynamic recovery, which leads to an increase in intragranular local stresses. Consequently, the choice of a specific strain rate must be determined by a compromise between the required grain size and the permissible level of internal stresses.

Summarizing the obtained data, it can be stated that a heating temperature of 650 °C is the thermomechanical optimum for the Zircaloy-4 alloy. It ensures high technological plasticity and structural stability, and it activates grain fragmentation mechanisms, paving the way for the effective production of UFG structures using plastic deformation methods without the risk of phase or thermal degradation of the material.

## 5. Conclusions

Based on the comprehensive plastometric, dilatometric, and microstructural investigations of the thermomechanical behavior of the Zircaloy-4 alloy, the following main conclusions were drawn:

1. It was established that the optimal temperature regime for the plastic deformation of the Zircaloy-4 alloy is the warm deformation region, specifically the temperature of 650 °C. Under these conditions, the deformation resistance decreases by approximately 70% compared to processing at room temperature. Concurrently, a minimization of the strain rate sensitivity of the flow stress is observed (convergence of the rheological curves for rates of 0.5 and 15 s^−1^), which is of key importance for ensuring the homogeneity of the stress–strain state during the deformation of massive cylindrical billets.
2. Based on the results of the dilatometric analysis, it was proven that heating to 650 °C guarantees the preservation of the stable initial α-phase structure of the alloy. The processes of secondary phase dissolution and the polymorphic α→β transformation are initiated at higher temperatures (above 694 °C and 847 °C, respectively), which completely eliminates the risk of uncontrolled phase degradation of the material during thermomechanical processing.
3. EBSD analysis demonstrated that plastic deformation at 650 °C leads to the intensive fragmentation of primary grains and the formation of a developed substructure with a high dislocation density. The primary mechanism of structural evolution is dynamic recovery combined with the initial stages of continuous dynamic recrystallization. An analysis of the grain size distribution confirms the influence of the kinetic factor on microstructural evolution: despite the same minimum recorded grain size (about 0.32 μm), high-rate deformation (15 s^−1^) leads to the formation of a coarser structure with a maximum grain size of up to 10.39 μm and a predominant grain area fraction at 5.0 μm. Decreasing the rate to 0.5 s^−1^ ensures the formation of a finer microstructure with a maximum size of 8.6 μm. The resulting state, with its high accumulated energy, serves as an effective precursor for obtaining ultrafine-grained (UFG) structures. High-temperature deformation (950–1050 °C), in contrast, leads to the undesirable formation of a coarse lamellar structure.
4. The nature of the strain rate’s influence within the optimal temperature window was revealed. It was established that decreasing the strain rate to 0.5 s^−1^ promotes the formation of a finer and more homogeneous grain structure, as the increased duration of the thermomechanical exposure provides the necessary conditions for a more complete progression of continuous dynamic recrystallization processes. Conversely, high-rate deformation (15 s^−1^) results in the formation of coarser grains.

## Figures and Tables

**Table 1:** Composition of Zircaloy-4 [28].

Element	Zr	Sn	Fe	Cr
Content, %	98.2	1.2–1.7	0.18–0.24	0.07–0.13

## Data Availability

The original contributions presented in this study are included in the article/Supplementary Material. Further inquiries can be directed to the corresponding author.

## Supplementary Materials

The following supporting information can be downloaded at: https://www.mdpi.com/article/10.3390/ma19091711/s1, Figure S1: Strain hardening curves of the Zircaloy-4 alloy during compression tests on the Gleeble 3800 plastometer; Figure S2: Inverse pole figure (IPF) orientation maps, kernel average misorientation (KAM) maps, and direct pole figures of the Zircaloy-4 alloy in the initial state and after deformation at various temperatures at a strain rate of 0.5 s^−1^; Figure S3: Inverse pole figure (IPF) orientation maps, kernel average misorientation (KAM) maps, and direct pole figures of the Zircaloy-4 alloy in the initial state and after deformation at various temperatures at a strain rate of 15 s^−1^.
