# Supplementary figures and images for: Study of the Influence of Thermomechanical Treatment on the Structure and Properties of Zircalloy-4 Alloy
Source: Materials (Basel). 2026 Apr 23;19(9):1711. doi: 10.3390/ma19091711 (PMC13164429; doi:10.3390/ma19091711)

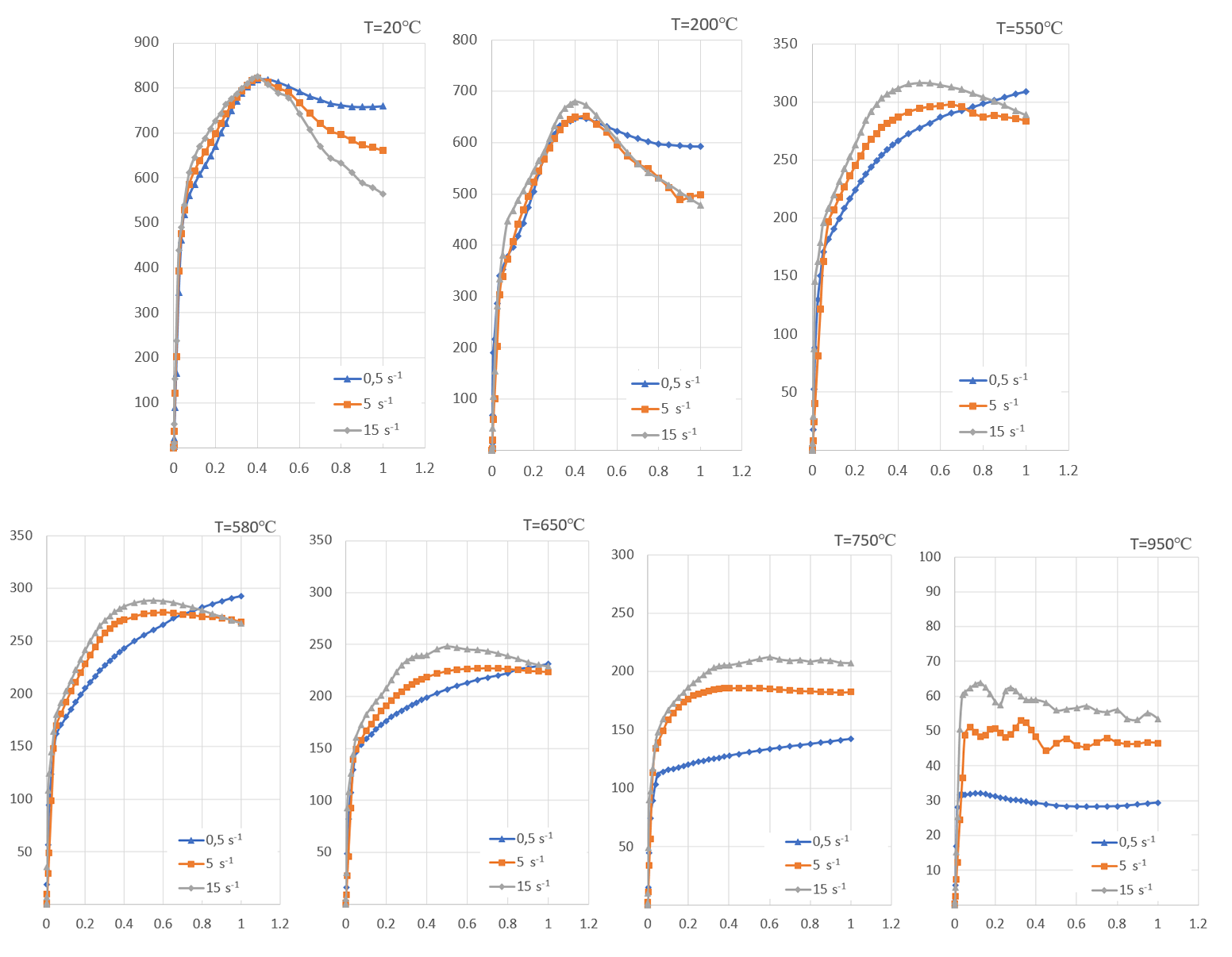

Supplement: Supplementary file 1 [file materials-19-01711-s001.zip › Figure S1.png]

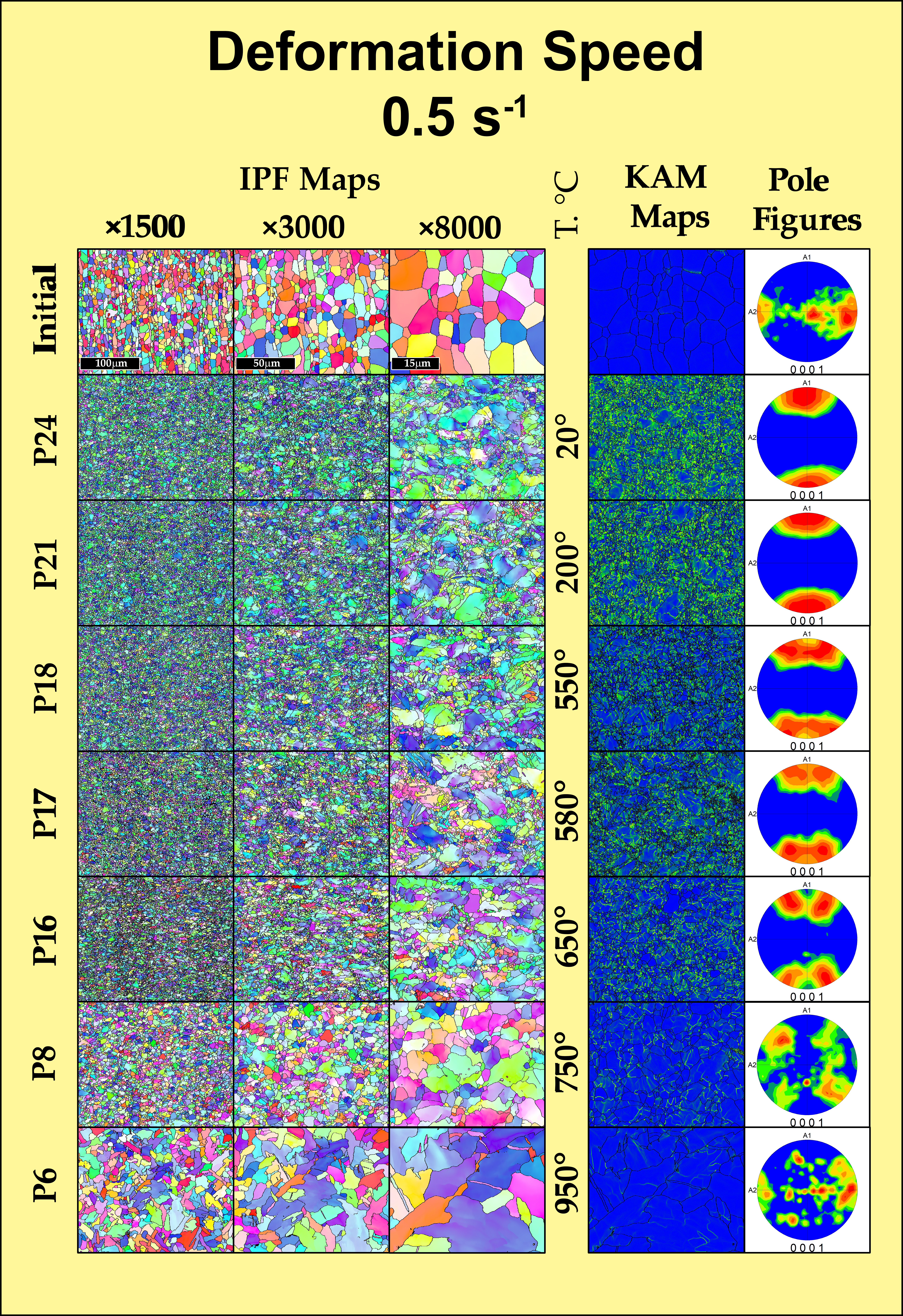

Supplement: Supplementary file 1 [file materials-19-01711-s001.zip › Figure S2.png]

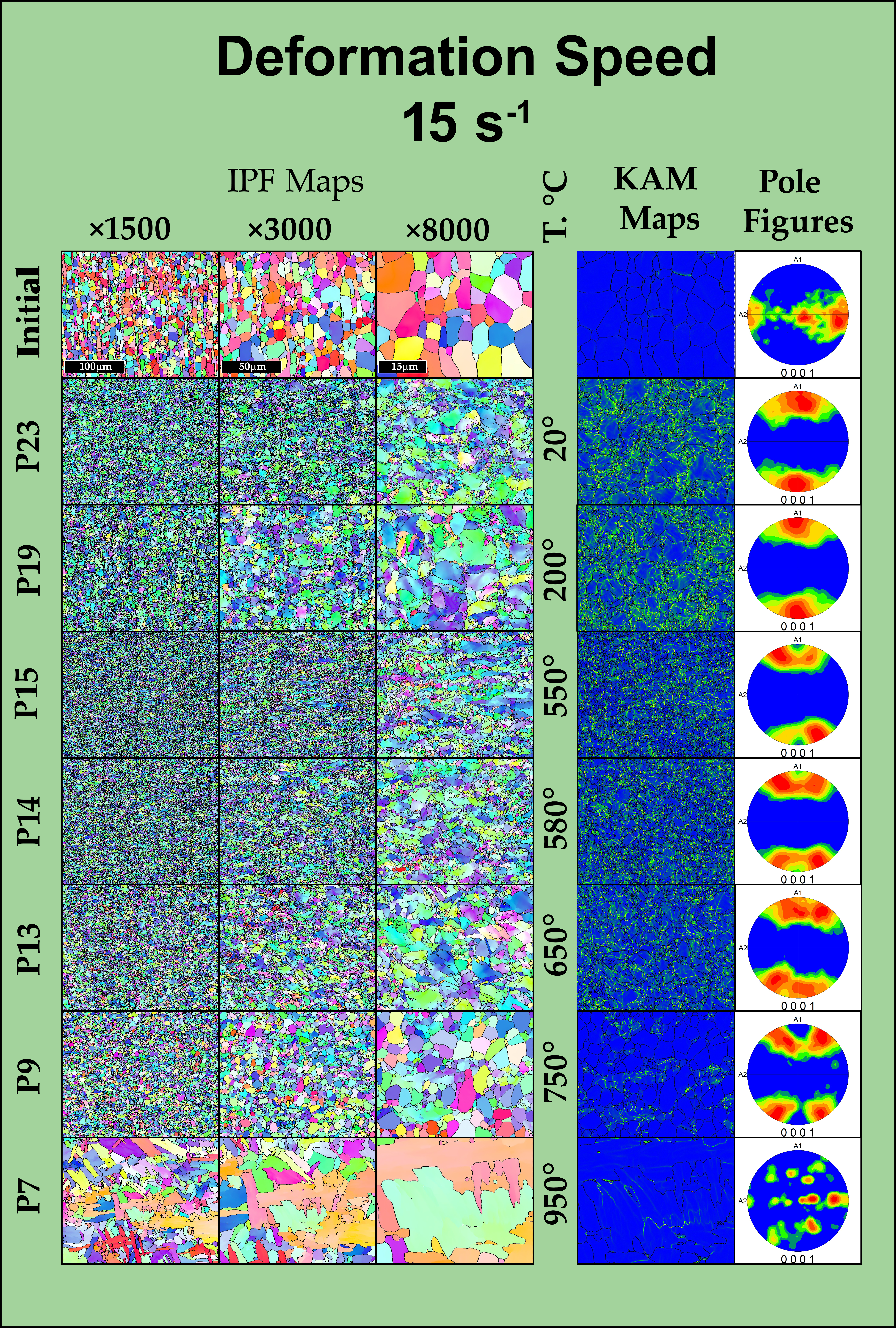

Supplement: Supplementary file 1 [file materials-19-01711-s001.zip › Figure S3.png]
